# *PqsA* mutation-mediated enhancement of phage-mediated combat against *Pseudomonas aeruginosa*

**DOI:** 10.3389/fcimb.2024.1296777

**Published:** 2024-02-26

**Authors:** Qiao Su, Di Lu, Jiuna Kong, Hong Lin, Guanhua Xuan, Jingxue Wang

**Affiliations:** State Key Laboratory of Marine Food Processing & Safety Control, College of Food Science and Engineering, Ocean University of China, Qingdao, China

**Keywords:** *P. aeruginosa*, pqsA, resistance, phage, enhancement of phage therapy

## Abstract

Phage therapy is a potential approach in the biocontrol of foodborne pathogens. However, the emergence of phage resistance and the narrow host range of most phage isolates continue to limit the antimicrobial efficacy of phages. Here, we investigated the potential of the *pqsA* gene, encoding the anthranilate-CoA ligase enzyme, as an adjuvant for phage therapy. The knockout of the *pqsA* gene significantly enhanced the bactericidal effect of phages vB_Pae_QDWS and vB_Pae_S1 against *Pseudomonas aeruginosa*. Under phage infection pressure, the growth of the PaΔ*pqsA* was significantly inhibited within 8 h compared to the wild-type PAO1. Furthermore, we found that altering phage adsorption is not how PaΔ*pqsA* responds to phage infection. Although *pqsA* represents a promising target for enhancing phage killing, it may not be applicable to all phages, such as types vB_Pae_W3 and vB_Pae_TR. Our findings provide new material reserves for the future design of novel phage-based therapeutic strategies.

## Introduction

*Pseudomonas aeruginosa* (*P. aeruginosa*) is an opportunistic pathogen that commonly causes spoilage in various vegetables, milk, and meat products (Garedew et al., 2012; Alimi et al., 2022; Gao et al., 2023; Wu et al., 2023). It has the ability to form biofilms, which provide protection against physical and chemical eradication methods, making it a significant concern for foodborne diseases and spoilage (O'Toole et al., 2000; Ma et al., 2022; Li et al., 2023). The global prevalence of multidrug-resistant (MDR) *P. aeruginosa* strains has become a serious threat to public health (Horcajada et al., 2019). In human beings, *P. aeruginosa* is responsible for a wide range of infections with varying levels of severity. *P. aeruginosa* infections can lead to pneumonia, urinary tract infections, bloodstream infections, wound infections, and respiratory tract infections (Bassetti et al., 2018; De Sousa et al., 2021). Therefore, precise and efficient prevention and control of *P. aeruginosa* infection is of great significance in ensuring food safety and quality, reducing the incidence of foodborne diseases, and protecting public health.

Bacteriophages, which are viruses that specifically infect bacteria, possess significant potential for selectively targeting and combating harmful bacteria (Kortright et al., 2019; Joao et al., 2021; Strathdee et al., 2023). Phages are naturally found in food and their effectiveness as powerful antimicrobials has been extensively documented (Guenther et al., 2009; Goodridge and Bisha, 2011; Sarhan and Azzazy, 2015; Kauppinen et al., 2021). The European Food Safety Authority (EFSA) has evaluated phages and concluded that they are safe for both consumers and the environment, although each phage or phage cocktail intended for use in food must be assessed on a case-by-case basis (Ricci et al., 2017; Rendueles et al., 2022). However, phage therapy can be complicated by the ability of bacteria to defend against phage attacks through various antiviral mechanisms, including spontaneous mutations, DNA restriction-modification, abortive infection systems, and the CRISPR-Cas adaptive immunity system (Labrie et al., 2010; Dupuis et al., 2013; Monteiro et al., 2019; Xuan et al., 2022). Overcoming phage resistance is a key issue that urgently needs to be addressed to enhance the effectiveness of phage therapy.

Several studies have focused on this issue and proposed diverse strategies to enhance the effectiveness of phage therapy. For example, phage genomes can be constructed using synthetic DNA fragments to introduce specific genetic modifications to enhance the efficacy of phage therapy (Lenneman et al., 2021). Researchers have also utilized phages as carriers to deliver biofilm-depolymerases, capsule-depolymerases, quorum-quenching enzymes, and cross-genus cell wall hydrolases with lytic activity, thereby enhancing the antimicrobial activity of phages (Pei et al., 2014; Born et al., 2017; Kilcher et al., 2018). Based on the temperate phage ΦCD24-2, Selle et al. (Selle et al., 2020) engineered a modified version that converted the phage’s lifestyle from temperate to virulent using genomic deletions, and simultaneously delivered host-targeting crRNA as a toxin, resulting in significantly enhanced phage-killing efficacy *in vitro* and in a *C. difficile* mouse infection model.

PqsA, an enzyme belonging to the CoA-ligase family, functions as the primary synthase in the biosynthetic pathway of alkyl quinolone (AQ) (Coleman et al., 2008; Witzgall et al., 2017). PqsA plays a pivotal role in regulating the production of virulence factors. Studies have shown that mutations in the *pqsA* gene significantly reduce *P. aeruginosa*’s ability to produce several virulence factors, including pyocyanin and elastase, which are key factors that contribute to *P. aeruginosa* pathogenicity (Bala et al., 2014). Thus, PqsA is often considered as a promising therapeutic target for mitigating or eradicating the virulence of *P. aeruginosa* (Ji et al., 2016; Shaker et al., 2020; Chen et al., 2022). While there is a research foundation for studying the functionality of PqsA protein and screening for inhibitors, its application in combating *P. aeruginosa* infections remains limited, and there is an urgent need to develop more effective treatment options such as phage therapy. However, phage therapy still faces the challenge of bacterial resistance to phage (Labrie et al., 2010; Dupuis et al., 2013; Monteiro et al., 2019; Xuan et al., 2022). Therefore, we initially focus on exploring the potential application of PqsA protein in enhancing phage therapy and identifying essential gene targets for designing phage-antibacterial agents combination therapy to effectively combat *P. aeruginosa* infections. However, there is scarce evidence on the significance of *pqsA* as a crucial gene target in phage therapy for combating *P. aeruginosa*.

In this study, we reported that mutations in the *pqsA* gene make *P. aeruginosa* less likely to develop resistance to phage infection in a short period of time, thereby enhancing phage sterilization. PqsA is expected to act as a new target for designing efficient phage therapy for the control of *P. aeruginosa*.

## Materials and methods

### Strains, plasmids, and growth conditions

*P. aeruginosa* strains were cultured under standard conditions at a temperature of 37°C in Luria Bertani broth (LB). Gentamicin and tetracycline were added at concentrations of 30 μg/mL and 50 μg/mL, respectively, for strain construction and plasmid maintenance purposes. Phages specific for *P. aeruginosa* PAO1 were isolated from sewage samples collected in Qingdao, China. The *Pseudomonas* phages, vB_Pae_S1 (accession number OL802210.1), vB_Pae_QDWS (accession number MZ687409.1), vB_Pae_W3 (accession number OK094665.1), and vB_Pae_TR (accession number OL802211.1) have been sequenced and deposited in NCBI GenBank.

### Gene knockout

The PaΔ*pqsA* strain was generated following a previously described protocol (Xuan et al., 2021). To construct a *pqsA*-deletion mutant, a 1047-bp fragment and a 1018-bp fragment upstream and downstream of *pqsA* were PCR-amplified from *P. aeruginosa* PAO1 genomic DNA. The two PCR products were fused together and cloned into pK18mobsacBtet plasmid at the EcoRI site. The constructed plasmid was transformed into *E. coli* S17–1 and then transferred via conjugation into *P. aeruginosa* PAO1. Integration into the chromosome of PAO1 was achieved through the first crossover event, followed by selection on tetracycline-containing agar plates with a chemically defined medium that solely utilized sodium gluconate as the carbon source. The double crossover was selected using 12% sucrose, resulting in the *pqsA*-deletion mutant. The colony PCR and DNA sequencing were performed to confirm the correct mutant.

### Phage resistance assay

Spot assay. A mixture of 100 μL of bacterial cultures (approximately 1.4 ×10^9^ CFU/mL) was added to 5 mL of 0.75% molten agar and poured onto the prepared LB plate. Subsequently, 3 μL of the phage suspension with serial dilutions was added onto the double-layer agar containing bacterial suspension. The plates were then incubated at 37°C without agitation. After incubation, the plates were examined for the presence of clearing zones.

The bacterial growth reduction assay. *P. aeruginosa* strains were co-cultivated with phage vB_Pae_S1 or vB_Pae_QDWS at a multiplicity of infection (MOI) of 0.1 at 37°C (The initial ratio of phage concentration to bacterial concentration is 1 × 10^7^ PFU/mL: 1.0 × 10^8^ CFU/mL). Bacteria cells were collected at different time points and the absorbance values at OD_600nm_ were measured and recorded using a microplate reader in a 96-well plate.

The efficiency of plating (EOP) assay. 3 μL of the phage suspension at various dilutions (10^5^, 10^4^, 10^3^, and 10^2^ PFU/mL) were applied to the surface of *P. aeruginosa* strains. The plates were then incubated at 37°C for 4 h. After incubation, the number of plaque-forming units (PFUs) was counted. The relative efficiency of plating (EOP) was calculated by dividing the average PFU count on the target bacteria by the average PFU count on the control PAO1 bacteria.

### One-step growth curve

The one-step growth curve experiment was conducted with some modifications following previously described methods (Xuan et al., 2023b). Briefly, *P. aeruginosa* cells were exposed to isolated phage at an MOI of 0.01 and allowed to adsorb for 3 min (for vB_Pae_QDWS) or 5 min (for vB_Pae_S1) at a temperature of 37°C. The mixture was then centrifuged at 10,000 × g for 1 min, and the resulting pellets were washed three times using LB medium. The supernatant was removed, and the pellets containing the phage-infected bacterial cells were resuspended in 25 mL of fresh LB broth. The suspension was incubated with shaking at 180 rpm and 37°C. Throughout the incubation period, samples were collected at specific time intervals, and the titers of the phage in the aliquots were immediately determined using the double-layer agar method.

### Phage killing assay

The overnight cultures of PAO1 and PaΔ*pqsA* were diluted 1:100 and cultured in fresh Casein Soya Bean Digest Broth (TSB) liquid medium to obtain a final bacterial concentration of approximately 0.4-0.6. Then, the bacterial cultures were subjected to a dilution with TSB liquid medium to achieve a final bacterial concentration of about 10^4^ CFU/mL. Simultaneously, the different multiplicity of infections (0.001 and 0.01) of phage vB_Pae_QDWS or vB_Pae_S1 were added to the TSB medium. The cultures were incubated at 37°C with shaking, and samples were collected at 0 h, 2 h, 4 h, 8 h, and 12 h time points. The cells were harvested by centrifugation and washed twice with phosphate-buffered saline (PBS). Subsequently, the cell counts of PAO1 and PaΔ*pqsA* infected with phage vB_Pae_QDWS or vB_Pae_S1 were determined at each time point.

### Host range analysis

Thirteen *P. aeruginosa* isolates were used to test the infectivity of phage vB_Pae_S1, vB_Pae_QDWS, vB_Pae_W3, and vB_Pae_TR. To determine the infectivity of the phages on the bacterial strains, a total of 3 μL of phage lysate with a titer of 10^11^ plaque-forming units (PFU) was applied onto an agar plate containing *P. aeruginosa* mixed with 0.5% (w/v) top agar. The plate was then incubated overnight. The infectivity of the phages was assessed by evaluating the turbidity of the plaques formed at the location where the phage lysate was dropped.

### Transmission electron microscope (TEM) analysis

Phage adsorption was observed by TEM as described previously (Xuan et al., 2023a). Briefly, *P. aeruginosa* cells were cultivated until reaching an optical density at 600 nm (OD_600nm_) of 2.5. Subsequently, the cells were mixed with phages vB_Pae_QDWS and vB_Pae_S1 at a multiple infection index (MOI) of approximately 100. After a 5 min (for vB_Pae_S1) or 3 min (for vB_Pae_QDWS) adsorption period, the samples were analyzed using TEM. The specific procedure for TEM analysis involved loading the samples onto a carbon-coated copper grid for 5 min, followed by negative staining with 2% (w/v) phosphotungstic acid (PTA, a common reagent in histological staining, pH 6.8). After drying, the samples were examined using a JEM-1200EX transmission electron microscope (JEOL, Tokyo, Japan) operating at 100 kV.

### Adsorption rate assay

Overnight cultures of *P. aeruginosa* strains PAO1 and PaΔ*pqsA* were diluted 1:100 and cultured in fresh LB medium until reaching an OD_600nm_ of approximately 0.4-0.6. To promote phage adsorption, 0.5 mL of a phage solution (10^5^ PFU/mL) was mixed with 0.5 mL of the cell suspension (10^8^ PFU/mL) and incubated at 37°C for 5 min (for vB_Pae_S1) or 3 min (for vB_Pae_QDWS). As a control, LB broth mixed with phage vB_Pae_S1 or vB_Pae_QDWS without bacteria was used. Following incubation, the cultures were centrifuged at 7,378 × g for 2 min, and the titer of free phage in the supernatant was determined using the double-layer agar method. The phage adsorption rate was calculated as follows: adsorption rate (%) = [(initial phage titer - phage titer in the supernatant)/(initial phage titer)] × 100.

### Bioinformatics analysis

The ViPTree (Nishimura et al., 2017) service (https://www.genome.jp/viptree/) was used to analyze the similarities and relationships between vB_Pae_QDWS and other reported prokaryotic double-stranded DNA viruses. A total of 3080 phage genomes were used as reference sequences to construct phylogenetic trees using VipTree. Sequence alignment of the whole genomes of four *Pseudomonas* phages was visualized using VipTree software.

## Results and discussion

### Disruption of *pqsA* of PAO1 could promote phage infection

**Figure 1:**
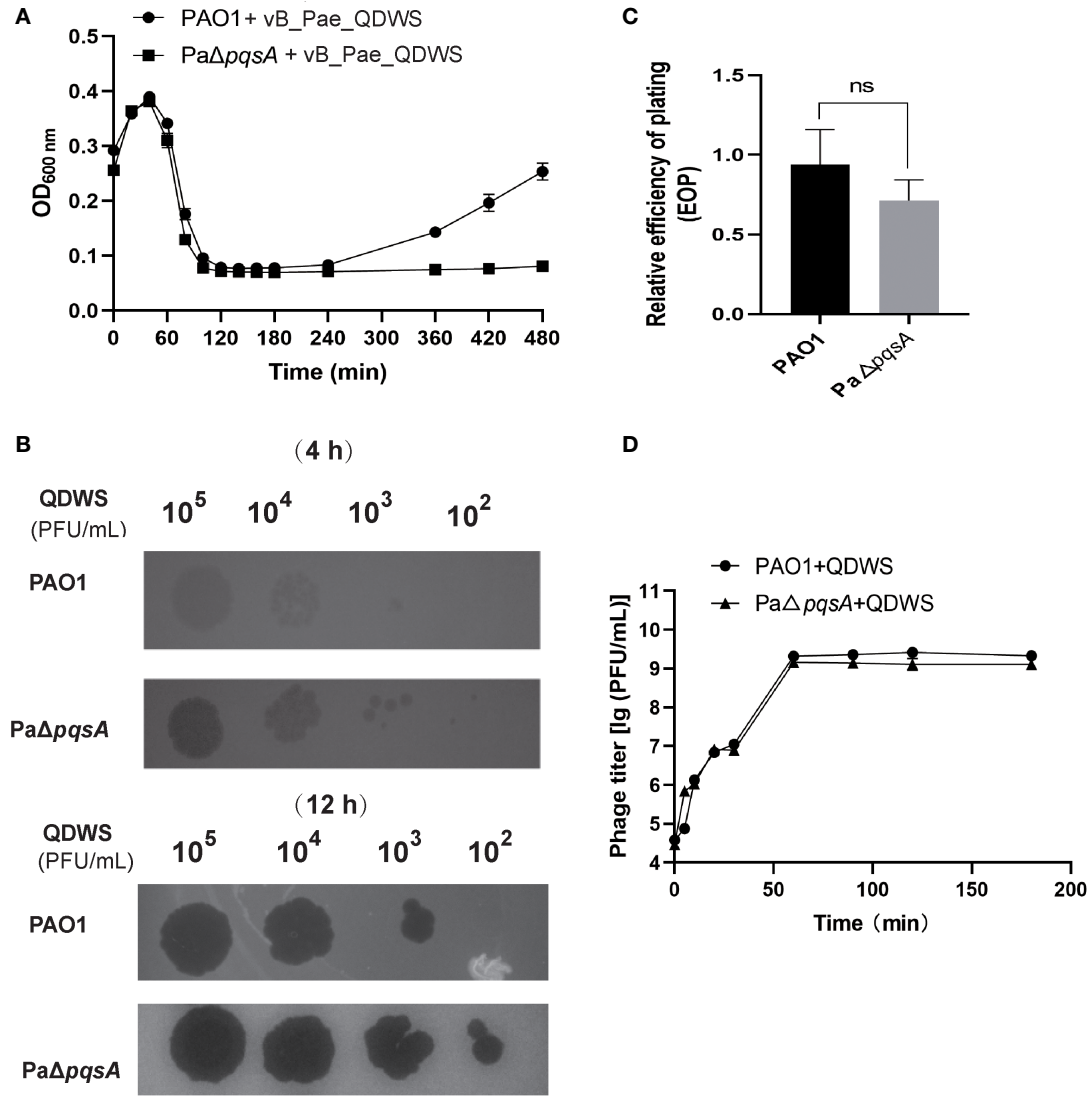
Knockout of the *pqsA* gene facilitates phage vB_Pae_QDWS infection of the host *P. aeruginosa*. **(A)** The growth curves of PAO1 and PaΔ*pqsA* strains, when infected with phage vB_Pae_QDWS, were measured at various time points. The samples were monitored by measuring the optical density (OD) at 600 nm using a SynergyH1 microplate reader in a 96-well plate. **(B)** Spot assays were conducted by spotting 3 μL of serial dilutions of phage vB_Pae_QDWS onto PAO1 and PaΔ*pqsA.* **(C)** Relative EOP of phage vB_Pae_QDWS on *P. aeruginosa* strains. Unpaired t tests were performed (‘ns’, not significant). **(D)** One-step growth curve determination of phage vB_Pae_QDWS on their respective host PAO1 and PaΔ*pqsA*. Mean values ± standard deviation were calculated from three independent experiments.

Here, we evaluated the effect of *pqsA* deletion on the survival of *P. aeruginosa* under the predation pressure of vB_Pae_QDWS. In the bacterial growth reduction assay, we found that phage vB_Pae_QDWS significantly reduced the cell count of *P. aeruginosa.* However, with the passage of time, a slow growth of PAO1 was observed after 240 min. Nevertheless, the growth of PaΔ*pqsA* remained significantly inhibited (**Figure 1A**). In different time intervals, the spot test findings also indicate that PaΔ*pqsA* is more prone to phage vB_Pae_QDWS infection, leading to the formation of more translucent plaques (**Figure 1B**). However, there was no significant difference in EOP of vB_Pae_QDWS on PAO1 and PaΔ*pqsA* strains (**Figure 1C**). Furthermore, growth curve analysis showed similar trends in one-step growth curves of the vB_Pae_QDWS using PAO1 and PaΔ*pqsA* as hosts (**Figure 1D**).

**Figure 2:**
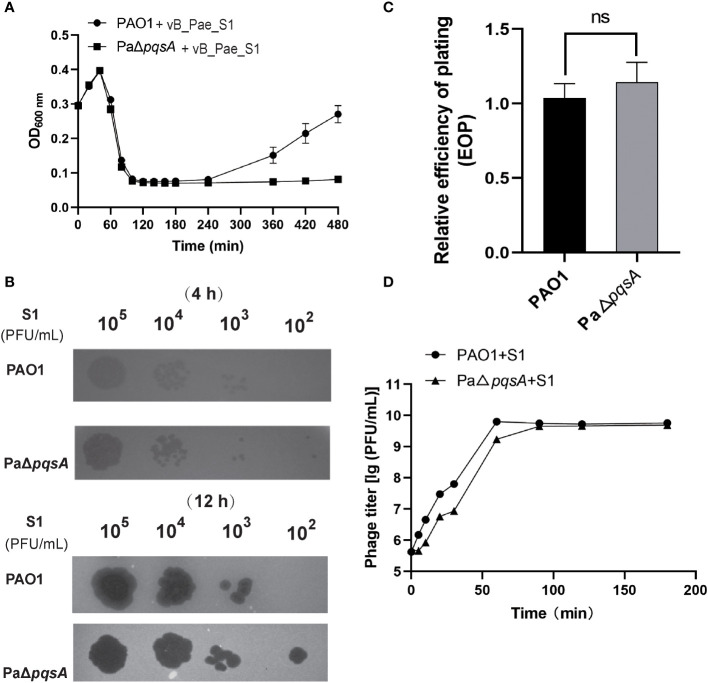
Knockout of the *pqsA* gene facilitates phage vB_Pae_S1 infection of the host *P. aeruginosa*. **(A)** The growth curves of the PAO1 and PaΔ*pqsA* strains were monitored at different time intervals upon infection with phage vB_Pae_S1. The optical density (OD) at 600 nm was measured using a SynergyH1 microplate reader in a 96-well plate. **(B)** Spot assays were performed by spotting 3 μL of serially diluted phage vB_Pae_S1 onto both PAO1 and PaΔ*pqsA*. **(C)** Relative EOP of phage vB_Pae_S1 on *P. aeruginosa* strains. Unpaired t tests were performed (‘ns’, not significant). **(D)** One-step growth curve determination of phage vB_Pae_S1 on their respective host PAO1 and PaΔ*pqsA*. Mean values ± standard deviation were calculated from three independent experiments.

Similar to the findings with phage vB_Pae_QDWS, we observed that phage vB_Pae_S1 also exhibited significantly higher infectivity towards the knockout strain PaΔ*pqsA* compared to the wild-type PAO1. Stronger growth inhibition after 240 min (**Figure 2A**) and more translucent plaques (**Figure 2B**) formed by the phage vB_Pae_S1 on the *pqsA* knockout strain during the phage infection assays were observed. However, there was no significant difference in EOP of vB_Pae_S1 on PAO1 and PaΔ*pqsA* strains (**Figure 2C**). Furthermore, growth curve analysis showed similar trends in one-step growth curves of the vB_Pae_S1 using PAO1 and PaΔ*pqsA* as hosts (**Figure 2D**). These results indicated that the knockout of the *pqsA* gene can enhance phage bactericidal efficacy. Our findings unveil a paradigmatic phage-bacteria interaction mediated by the *pqsA* target. This discovery provides crucial insight for further research and development of therapeutic strategies harnessing the phage-bacteria interaction, particularly through targeted silencing of the *pqsA* gene to enhance the precision of phage therapy against *P. aeruginosa*. This advancement contributes to filling the research gap in the field of precise pathogen control.

**Figure 3:**
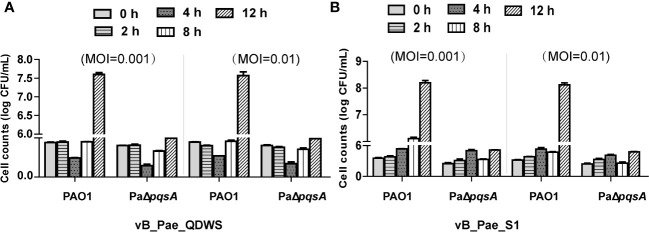
The knockout of the *pqsA* gene of *P. aeruginosa* can enhance the efficacy of phage therapy. The ability of phages **(A)** vB_Pae_QDWS or **(B)** vB_Pae_S1 to lyse *P. aeruginosa* PAO1 and PaΔ*pqsA* strains at different multiplicity of infections (0.001 and 0.01) in TSB medium at 37°C. Cell counts of PAO1 and PaΔ*pqsA* infected with phage vB_Pae_QDWS or vB_Pae_S1 were detected at different time points. Mean values ± standard deviation were calculated from three independent experiments.

Phages vB_Pae_QDWS and vB_Pae_S1 had genome sizes of 43,170 and 43,058 bp, respectively. Both of them are lytic phages targeting *P. aeruginosa*. The two phages vB_Pae_QDWS and vB_Pae_S1 shared an intergenomic nucleotide identity of 98.39% (**Table 1**). Here, we explored the lytic abilities of phages vB_Pae_QDWS and vB_Pae_S1 against both PAO1 and PaΔ*pqsA* at MOI 0.001 and MOI 0.01. Phage vB_Pae_QDWS effectively suppressed the growth of both PAO1 and PaΔ*pqsA*, as there was no significant increase in bacterial cell numbers within 8 h. However, with prolonged co-cultivation, a noticeable growth acceleration of PAO1 was observed, while PaΔ*pqsA* maintained a lower cell count (**Figure 3A**). The results of the lysis assay for phage vB_Pae_S1 were similar to those of vB_Pae_QDWS (**Figure 3B**). Therefore, our data suggest that the knockout of the *pqsA* gene can enhance the efficacy of phage therapy.

**Table 1:** Comparative analysis of the genomic characteristics of vB_Pae_Q+.

	coverage (96%) vB_Pae_QDWS	identity (98.39%) vB_Pae_S1
Genome size (bp)	43,170	43,058
G+C (%)	62.3	62.22
tRNAs	0	0
Predicted ORFs	53	57

### Enhanced infection mediated by *pqsA* mutation is independent of adsorption

**Figure 4:**
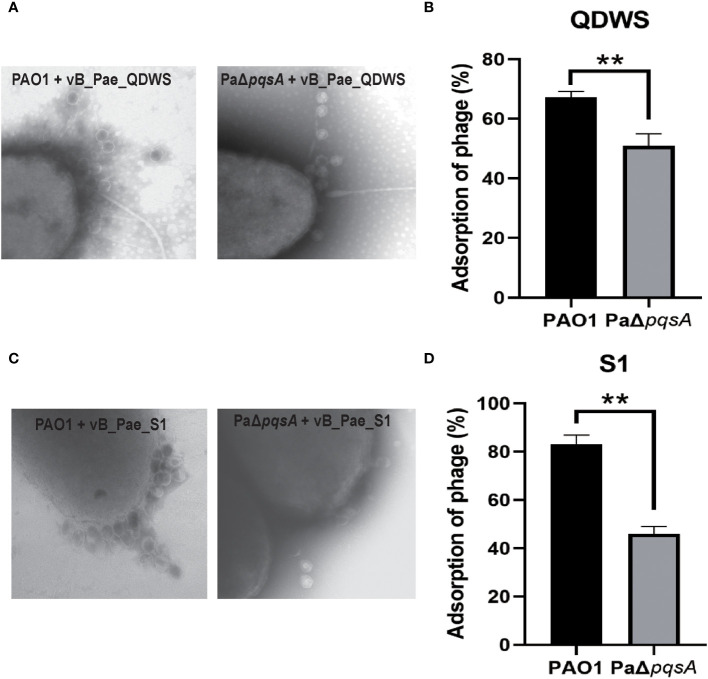
The enhanced phage infection mediated by *pqsA* mutation is independent of adsorption. **(A, B)** Adsorption assays of phage vB_Sb_QDWS to *P. aeruginosa* PAO1 and PaΔ*pqsA* strains. **(C, D)** Adsorption assays of phage vB_Sb_S1 to *P. aeruginosa* PAO1 and PaΔ*pqsA* strains. Mean values ± standard deviation were calculated from three independent experiments. A t-test was performed (**, P < 0.01).

Adsorption, as the primary step of phage invasion into the host, often affects the bactericidal efficacy of phages due to changes in their adsorption efficiency (Denes et al., 2015; Harvey et al., 2018). We conducted phage adsorption assays using the PAO1 and PaΔ*pqsA* strains. The results of TEM analysis revealed that a higher number of vB_Pae_QDWS phages were observed surrounding the PAO1 strain compared to the host PaΔ*pqsA* strain (**Figure 4A**). Additionally, we observed a decrease in the adsorption rate of phages to the PaΔ*pqsA* strain compared to the PAO1 strain (**Figure 4B**). Similar results were also observed for vB_Pae_S1, with significantly lower adsorption efficiency to PaΔ*pqsA* compared to its efficiency to PAO1 (**Figures 4C, D**). In theory, a decrease in the adsorption efficiency of phages would significantly reduce their chances of invading the host. However, PaΔ*pqsA* exhibited greater sensitivity to phages compared to PAO1 (**Figures 1**, **2**). These results supported that the enhanced sensitivity of the host to phages mediated by *pqsA* mutation is not attributed to the enhancement of phage adsorption pathway.

### Hypothesizing the mechanism of phage sensitivity mediated by *pqsA* mutation

Based on the experimental data mentioned above, we have reasons to speculate that *pqsA* mutation significantly inhibits the development of phage resistance. Literature reports have indicated that phase variation in receptor structure mediates the coexistence of phages and bacteria, maintaining a balance between host sensitivity and phage resistance (Shkoporov et al., 2021; Ramos-Barbero et al., 2023). Growth curve analysis of phage-host cocultures revealed that, after 240 min, the wild-type PAO1 strain exhibited significant growth, while PaΔ*pqsA* remained suppressed (**Figures 1A** **,** **2A** **,** **4B**), suggesting that the *pqsA* mutant strain might have lost the ability to regulate flexible phase variation in receptor structure in the face of peak phage predation pressure, leading to a deficiency in bacterial immunity against phages compared to the wild-type strain PAO1. The *pqsA* mutation does not affect phage infectivity, as evidenced by the similar phage burst efficiencies (**Figures 1C**, **2C**), as well as comparable growth curve trends of phage infection in the wild-type PAO1 and PaΔ*pqsA* (**Figure 1D**, **2D**). The *pqsA* mutation specifically impairs bacterial immunity against phages, likely mediated by changes in receptor structure phase variation, which is consistent with our observation that knocking out the *pqsA* gene significantly reduces phage adsorption efficiency (**Figure 4**). Although the *pqsA* gene mutation hinders the rapid development of phage resistance in the host, further research is needed to uncover and elucidate the involvement of PqsA in the regulation of phage-host interactions mediated by receptor phase variation. Future integration of transcriptomic analysis with phenotype association is expected to elucidate important pathways and mechanisms through which the changes in phage-bacteria interactions mediated by *pqsA* mutations.

### The multiple effects of silencing the *pqsA* gene on the application of phage therapy

**Figure 5:**
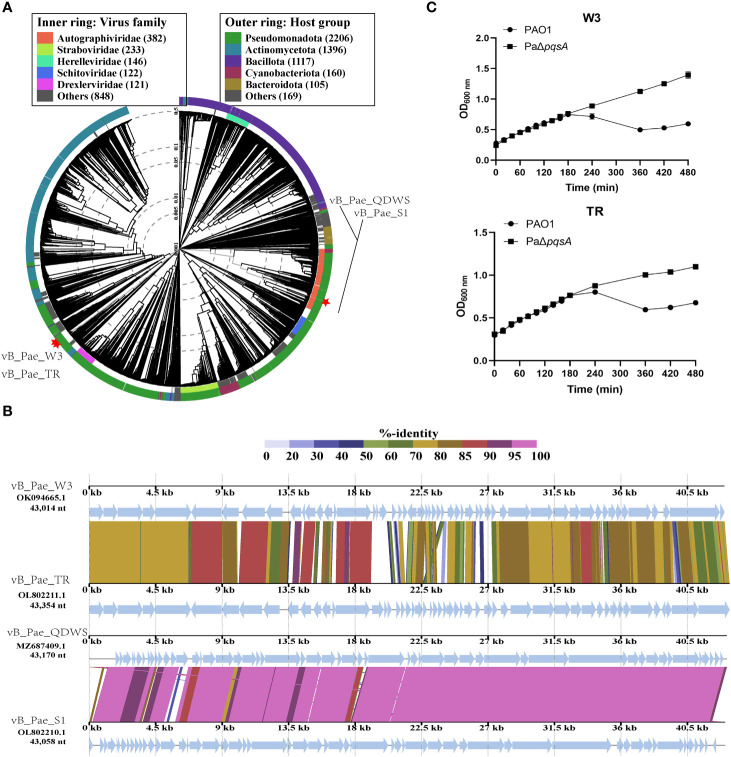
The response of PaΔ*pqsA* strain to the infection of phages vB_Pae_W3 and vB_Pae_TR. **(A)** ViPTree analysis of four *Pseudomonas* phages. Phages are identified according to their official ICTV classification, with the outer and inner rings indicating their host group and virus family, respectively. **(B)** Comparative genome alignment of the *Pseudomonas* phages vB_Pae_QDWS, vB_Pae_S1, vB_Pae_W3, and vB_Pae_TR. Analysis was performed using VipTree software. The color indicates the nucleotide sequence identity level (from 0 to 100%). **(C)** The growth curves of PAO1 and PaΔ*pqsA* strains, when infected with phage vB_Pae_W3 and vB_Pae_TR, were measured at various time points.

We also compared and investigated multiple *P. aeruginosa* phages (vB_Pae_QDWS, vB_Pae_S1, vB_Pae_W3, and vB_Pae_TR) to analyze the impact of silencing the *pqsA* gene on the bactericidal ability of the phages. As shown in **Figure 5A**, phage vB_Pae_QDWS and vB_Pae_S1 were classified into different branches from vB_Pae_W3 and vB_Pae_TR, indicating different divergences. Comparative analysis of four *P. aeruginosa* phages in BLASTn is given in **Figure 5B**. Phage vB_Pae_QDWS and vB_Pae_S1 displayed a close relationship, and vB_Pae_W3 had similarities with vB_Pae_TR. However, except for S1, QDWS shows no similarity to vB_Pae_W3 or vB_Pae_TR. In the bacterial growth reduction assay, we found that deletion of the *pqsA* gene leads to enhanced resistance of the host strain to phage vB_Pae_W3 and vB_Pae_TR. After 180 min, it was observed that the growth rate of PAO1 was significantly delayed compared to that of PaΔ*pqsA* (**Figure 5C**), which is quite different from the bactericidal effects observed during phage vB_Pae_QDWS and vB_Pae_S1 treatment. Therefore, silencing the *pqsA* gene leads to different outcomes in the interaction between the host and phages, which may be attributed not only to the regulation mediated by changes in the host’s genetic and metabolic networks but also to the type of phage involved.

### Host range analysis of four *Pseudomonas* phages

The efficiency of the lytic activity of four phages was measured against thirteen *P. aeruginosa* strains using the visual assessment of plaques on the spot test. Seven (53.8%) and six (46.2%) of the *P. aeruginosa* strains tested were lysed by vB_Pae_W3 and vB_Pae_TR, respectively, while phages vB_Pae_QDWS and vB_Pae_S1 exhibit similar lysis spectra, being able to lyse ten (76.9%) *P. aeruginosa* strains. Phages vB_Pae_QDWS and vB_Pae_S1 exhibit stronger lytic activity and a broader range of lysis compared to phages vB_Pae_W3 and vB_Pae_TR, making them appear to have a competitive advantage in combating *P. aeruginosa* (**Table 2**).

**Table 2:** Lytic activity of four *Pseudomonas* phages (+, infected; −, uninfected).

Strain	Source	*Pseudomonas* phages			
		vB_Pae_W3 vB_Pae_TR vB_Pae_S1 vB_Pae_QDWS			
*P. aeruginosa* PAO1	Standard strain	**+**	**+**	**+**	**+**
*P. aeruginosa* Y3	this study	**-**	**-**	**-**	**-**
*P. aeruginosa* Y4	this study	**+**	**+**	**+**	**+**
*P. aeruginosa* Y9	this study	**+**	**-**	**+**	**+**
*P. aeruginosa* Y14	this study	**+**	**+**	**-**	**-**
*P. aeruginosa* SJ-1	this study	**-**	**-**	**+**	**+**
*P. aeruginosa* SJ-2	this study	**-**	**-**	**+**	**+**
*P. aeruginosa* SJ-4	this study	**+**	**+**	**+**	**+**
*P. aeruginosa* SJ-6	this study	**+**	**+**	**+**	**+**
*P. aeruginosa* SJ-8	this study	**-**	**-**	**+**	**+**
*P. aeruginosa* SJ-10	this study	**-**	**-**	**+**	**+**
*P. aeruginosa* SJ-76	this study	**+**	**+**	**-**	**-**
*P. aeruginosa* PA14	Standard strain	**-**	**-**	**+**	**+**

## Conclusion

In this study, we show that *pqsA* has the potential to become an important gene target to enhance phage therapy. The deletion of the *pqsA* gene could significantly promote the lysis of phages vB_Pae_QDWS and vB_Pae_S1 on the *P. aeruginosa* PAO1. We speculate that the mechanism may be related to the defect in bacterium-phage immune capability mediated by *pqsA* mutation, as we observed that *pqsA* mutation mainly affects the later stage of phage-host interaction, specifically inhibiting the development of phage-resistant strains. However, the specific regulatory mechanisms still require further research and clarification. Although silencing the *pqsA* gene is expected to enhance the efficacy of phage therapy against *P. aeruginosa*, it also depends on the specific phage type used. For example, silencing *pqsA* may significantly decrease the therapeutic effectiveness of phages vB_Pae_W3 and vB_Pae_TR. Regardless, we propose that the *pqsA* gene plays a crucial role in mediating the phage-bacteria interaction process. However, in future studies aiming to design novel phage therapies targeting the *pqsA* gene, further validation is required using a broader range of phage targets.

## Data availability statement

The datasets presented in this study can be found in online repositories. The names of the repository/repositories and accession number(s) can be found in the article/supplementary material.

## Author contributions

QS: Writing – review & editing, Conceptualization, Data curation, Methodology. DL: Writing – review & editing, Conceptualization, Data curation, Formal analysis, Methodology. JK: Conceptualization, Methodology, Formal analysis, Investigation, Writing – review & editing. HL: Conceptualization, Project administration, Supervision, Writing – review & editing. GX: Writing – original draft, Writing – review & editing. JW: Writing – review & editing.
